# Assessing the prevalence, sources and selective antecedents of organizational stressors among elite football players and coaches in the Ghana premier league: Empirical evidence for applied practice

**DOI:** 10.3389/fspor.2022.938619

**Published:** 2022-07-28

**Authors:** Medina Srem-Sai, John Elvis Hagan, Prosper Narteh Ogum, Thomas Schack

**Affiliations:** ^1^Department of Health, Physical Education, Recreation and Sports, University of Education, Winneba, Ghana; ^2^Department of Health, Physical Education and Recreation, University of Cape Coast, Cape Coast, Ghana; ^3^Neurocognition and Action-Biomechanics-Research Group, Faculty of Psychology and Sport Sciences, Bielefeld University, Bielefeld, Germany

**Keywords:** football coaches, Ghana, players, premier league, stress

## Abstract

Globally, job-related stress has been classified as a health epidemic which is common among many individuals across diverse populations. Despite this established knowledge, research has primarily focused on the general population and among health workers. Therefore, understanding stress related experiences in the context of professional sport would help design appropriate stress management interventions for effective coping. The overarching aim of this research was to assess occupational stress related experiences among players and coaches in the Ghana premier league. The study sought to assess: (1) the prevalence and sources of stressors among players and coaches, and (2) how age and years of experience influenced the stressors they experienced. Using a census survey, 44 premier league coaches and 424 players who were officially registered by 17 premier league clubs completed the intensity dimension of the Organizational Stressor Indicator for Sport Performers which has 5 subscales namely: Goals and Development, Logistics and Operations, Team and Culture, Coaching and Selection. Descriptive statistics (means and standard deviation) and multivariate analysis of variance were used to analyze the data. The results revealed that stressors were prevalent among football players and coaches, with these being significantly more dominant among coaches, *p* < 0.001. Selection was identified as the most predominant stressor for coaches (i.e., selecting players to play) and players (i.e., being selected). Age and experience were found not to be significant predictors of stressors for players and coaches in Ghana. Findings suggest that generally, stressors are common among football players and coaches, especially on issues related to selection. Sport psychologists and team managers in the various premier league clubs should incorporate appropriate interventions (e.g., stress inoculation training) aimed at providing adequate psychological support to promote players' and coaches' wellbeing.

## Introduction

Participating in professional sport places extreme demands on athletes and coaches in their quest to achieve a common goal (Mellalieu et al., 2009; Fletcher and Scott, 2010). Previous studies have indicated that elite athletes and coaches operate in a complex environment which exposes them to many stressful situations because of the demands put on them (Mellalieu et al., 2009; Fletcher and Scott, 2010; Olusoga, 2011; Kristiansen et al., 2012). In the present study, stress is defined as “a continuous process in which people transact with their environment, evaluate the situations they encounter, and strive to deal with matters that may occur” (Fletcher et al., 2006, p. 329). Stressors are also referred to as the environmental demands and/or stimuli put on a person either within his/her organization or competition environment (Fletcher et al., 2006). It is worth noting that when an individual experiences a stressor, that does not necessarily lead to stress unless the person is unable to manage the situation (Mellalieu et al., 2009; Fletcher and Scott, 2010; Arnold et al., 2013). General psychology literature has shown that individuals who encounter stressors that they cannot deal with experience many physical symptoms (e.g., sleeping difficulties, fatigue), psychological symptoms (e.g., anxiety, anger, depression, burnout) and behavioral symptoms like work drop-out, weeping and mood swings (World Health Organisation, 2016).

Within sport psychology, previous studies (e.g., Mellalieu et al., 2009; Fletcher and Scott, 2010; Kristiansen et al., 2012; Thelwell, 2012; Thelwell et al., 2016a,b) that examined stressors coaches and athletes experience have indicated diverse results. For example, Kristiansen et al. (2012) investigated a sample of US football players and found that inequities in the salary of players, lack of information about other teammates', and very low or high salaries were regarded as stressors to these athletes. The athletes in Kristiansen's study further acknowledged that unhealthy and intense rivalry for team placement negatively impacted the team's general morale and performance (Kristiansen et al., 2012). Additionally, Thelwell et al. (2008) found about 182 stressors experienced by coaches as organizational related on a wide range of issues such as training environment, selection, team atmosphere, competition environment, travel, finances, athletes, other coaches, communication, and roles that they play in their teams (Thelwell et al., 2008). Similarly, Mellalieu et al. (2009) identified performance-related stressors among elite athletes to be preparation, injury, expectations, opponents, coach issues, and self-presentation while organizational stressors included facilities, spectators, officials, format of competition, roles and other athletes. Within the context of Africa, Kubayi et al. (2018) in their validation of an instrument to examine stressors among South African coaches, identified four components of stressors experienced by these coaches as environmental stressors (e.g., poorly planned travel arrangements, long working hours, poor hygiene conditions, unsafe competition arena), performance stressors (e.g., being blamed for poor results, financial incentives dependent on results, high expectation to win), task-related stressors (e.g., performing multiple roles like selection and scouting, managing too many squads, managing other coaches in the program, lack of recognition for good coaching), and athlete stressors (e.g., conflict between me and my athletes, athletes under-performing in training, conflict between athletes, injury to one of my athletes). Again, Surujlan and Nguyen (2009) identified sources of stress among South African coaches to be lack of resources, external pressure and internal capacity as multiple pressures experienced by African coaches. According to stress researchers, if these stressors are not appropriately dealt with, they can negatively affect players' and coaches' emotions (Fletcher et al., 2012; Hagan et al., 2018), performance outcomes (Fletcher et al., 2006; Kristiansen et al., 2012; Lu et al., 2012; Fletcher and Arnold, 2016), and general psychological health and wellbeing (Cooper and Marshall, 2013; World Health Organisation, 2016).

Other studies have found that these stressors encountered by players and coaches are influenced or moderated by different antecedents (Nicholls and Polman, 2007). For example, Nicholls and Polman (2007) found a relationship between athletes' stressors, type of sport and skill levels in which players in team sports reported many stressors such as the environment surrounding their teams. Nicholls and Polman (2007) further revealed that highly skilled athletes reported coping better with stressors than their less skilled counterparts. Moreover, Frazer (2005) found that competitive level revealed no difference between NCCA coaches and those working at other levels. Additionally, age and years of experience have also proven to be associated with sport performers' experiences of stressful situations. For instance, Hagan et al. (2018) found that age and years of experience of sport performers considerably affected their experiences of competitive anxiety. For example, existing skill classification indicates the likelihood that an athlete who is regarded as highly skilled would have a very low experience because of a sudden increase in her or his sport performance (Hagan et al., 2018). Additionally, chronological age which is associated with competitive experience of athletes could also influence competitive anxiety (Hagan et al., 2018). There is therefore the possibility that more experienced or older athletes and coaches might be minimally affected by related anxiety symptoms more than less experienced or younger sport performers who might be skilled or otherwise (Hagan et al., 2018). Comparatively, Hammermeister and Burton (2001) found that older endurance athletes demonstrated lower cognitive anxiety than their younger colleagues due to the usage of fewer ego-threatening goals during competitive situations.

Even though there are several stress related studies in Ghana, it is surprising that stress research in the country has primarily focused on non-sporting populations such as security personnel (Gyamfi, 2014; Arthur, 2016), health workers (Acquaye, 2011), and other employees (Azumah, 2014; Duah, 2016; Nnuro, 2016). Arthur (2016), for example, investigated occupational stressors among police officers in the Central Region of Ghana and revealed organizational constraints like work overload, public criticisms, and accommodation as more stressful to the sampled police officers than their exposure to physical hazards. Similarly, Nnuro (2016) found workload as a major stressor among Polytechnic staff in the Eastern Region of Ghana. The fact that stress-related researchers in Ghana have ignored the sporting population (e. g., athletes and coaches) across diverse sport disciplines has created an empirical vacuum when relating to the specific stressors experienced by Ghanaian sport players and coaches. It is also not clear whether stressors encountered by football players and coaches in other continents or jurisdictions would apply to the Ghanaian context due to context-specific reasons. For instance, some studies have suggested the possibility of some stressors being peculiar or exclusive and unique to some particular cultural groups and contexts (Noblet and Gifford, 2002; Mckay et al., 2008). Further, specificities about culture would be necessary to provide understanding about the way other teams or groups perceive stressors (Kristiansen et al., 2012). Some scholars have also stressed the need for culturally diversified research and practice in sport psychology to create a better awareness regarding different cultures and their stress experiences in sport. This perspective is reiterated by Markus and Kitayama (1991) and Dzokoto (2010) who opined that psychological episode like stress could be idiosyncratic to specific context and not universal across cultures.

Therefore, this study examined the prevalence of stressors experienced by football players and coaches in the Ghana premier league, their sources, and how their ages and years of experience relate to the stressors they encounter. Empirical information gathered from this study would guide sport psychologists to design specific stress management interventions for football players and coaches. Additionally, the study would form a basis for future stress-related research among coaches, footballers, and other sporting populations in Ghana.

## Materials and methods

### Study participants

The descriptive cross-sectional survey design was adopted to select a total of four hundred and sixty-eight (468) male football players and coaches who participated in the 2020/2021 Ghana Premier League (GPL) season using census. Although a total of 594 participants undertook the survey (including 540 players and 54 coaches), 424 players and 44 coaches officially completed the survey, resulting in a response rate of 78.5 and 81.5%, respectively. The players' age ranged from 16 to 32 years (*M*_age_ = 22.36, *SD* = 3.53) while coaches' ages ranged from 31 to 70 years. Years of experience for players ranged from 1 to 15 years (*M* = 2.69, *SD* = 1.82) and 1 to 17 years for coaches. Majority of the participants reported years of experience below 5 years. The inclusion criteria required that the individual player or coach was officially registered by the Ghana Football Association who took part in the 2020/2021 Premier League season. In line with Swann's et al. (2015) classification of elite athletes, survey participants were regarded as elite because the Ghana premier League is considered to be the highest level of professional football in the country. Thus, reaching this level of competitiveness (premier level) is a great achievement in their professional career. Only 9 (2.12%) of the participants had diploma and degree certificates, while 143 (33.72%) were secondary school certificate holders. Majority of the participants (*n* = 272, 64.16%) were either junior high or primary school graduates.

### Instrumentation

#### Organizational stressor indicator for sport performers (OSI-SP)

Adopting the intensity dimension of the 23-item OSI-SP developed by Arnold et al. (2013), football players' and coaches' experiences of organizational stressors were assessed during the 2020/2021 GPL season. Some preliminary information on the instrument informed participants to be open and honest, and asked them to respond to the survey instrument according to the team they mostly played for, in case they had played for two teams or more within the season. The instrument has five-subscales including: Team and Culture (four-items; e.g., “the shared beliefs of my teammates”), Goals and Development (six-items; e.g., “the development of my sporting career”), Logistics and Operations (nine-items; e.g., “my training schedule”), Selection (two-items; e.g., “how my team is selected”) and Coaching (two-items; e.g., “the relationship between my player/coach and I”). Every item on the instrument had the stem “In the past month, I have experienced pressure associated with,” to which every participant answered on only the intensity dimension of the scale ranging from 0 to 5. The intensity scale was “how demanding was this pressure?” where 0 represents no demand; 1, very low demand; 2, low demand; 3, moderate demand; 4, high demand; and 5, very high demand. Based on this categorization, a mean score below 2.45 denoted low demand, between 2.45 and 3.44 represented moderate demand and >3.44 signified high demand as explained on the scoring sheet of the instrument by Arnold et al. (2013). Previous studies reported internal consistency values using Cronbach's alpha coefficients for the intensity scale ranging from 0.50 to 0.83 (Arnold et al., 2013; Srem-Sai et al., 2021). The current study reported Cronbach alpha coefficient values from 0.66 to 0.83.

#### Pre-testing of the instrument

The instrument (OSI-SP) was pre-tested using football players and coaches who took part in the 2020/2021 Division 1 football league which is just one step below the premier league level. This pre-testing was done to check for homogeneity of characteristics of the main sample. It also helped to identify lapses in advance and areas where the instrument may need some adjustment. This enabled the researchers to adjust the instrument to make it suitable for both players and coaches to respond to without any confusion. For example, the item which states, “the relationship between my coach and I” was adjusted as “the relationship between my coach/player and I.”

### Procedure

Following ethical approval by the Institutional Review Board of the University of Cape Coast (IRB-UCC) to conduct the study with reference number UCCIRB/CES/2020/42, additional permission was sought from organizers of the league, owners of the teams and their managers. The participants' recruitment process began by meeting with Chief Executive Officers (CEOs) of the various teams, team managers, players and coaches to discuss the rationale of the research. While familiarization was going on, the survey instrument was thoroughly explained to the participants who were further briefed about their rights to withdraw from being part of the study, right to keeping every response they provide anonymous and keeping their responses confidential. Participants were also assured that the information provided would be accessible to the researchers only.

Thirty-four (34) research assistants (two from each team) were selected and trained by the researchers to help in gathering the data. This approach was used after considering the geographical settings and the specific language/dialect spoken. Regarding the two assistants for each team, one should be fluent in English Language while the other was supposed to be fluent in the native Language/dialect based on their geographical location. The research assistants were thoroughly briefed about the rationale of the study while each item on the survey instrument including the scales were well explained to them. Data collection started four (4) weeks after the 2020/2021 premier league season began because participants were supposed to relate to stressors/demands they experienced “one month ago” as indicated on the survey instrument. Prior to gathering the data, each participant endorsed an informed consent form after which the survey instruments and pencils were distributed to them by the trained research assistants to respond to at their home grounds after morning training sessions. Providing answers to the questionnaires took about 15–20 min which lasted for about a period of 3 months for all the teams. Participants who could not answer the questionnaire because of language barriers were assisted by the research assistants using their local dialects. All COVID-19 safety protocols were observed during all face-to-face administration of the instrument. All answered survey instruments were retrieved in sealed brown envelops.

### Data analysis

Data was statistically tested for multivariate linearity and outliers, missing data, univariate and multivariate normality, multicollinearity, and variance-covariance matrix homogeneity. For univariate and multivariate normality, Q-Q plots were used to check whether all data points were closer to the line. To check for linearity assumptions and outliers, scatter plots were used. The Box's *M* test of equality of covariance matrices was used to assess the homogeneity of variance-covariance, and multicollinearity assumptions were also examined to establish how the dependent variables were connected. These were done to minimize errors in the actual data to be analyzed. After a successful screening was done, the IBM SPSS statistical software version 22 was used to analyse the screened data. Participants' demographic information (age and years of experience) and the organizational stressors prevalent among football players were assessed using means and standard deviations. Further, to investigate the relationship between organizational stressors and participants' ages and years of experience, the factorial Multivariate Analysis of Variance (MANOVA), with age and years of experience as independent variables and the five (5) dimensions of the OSI-SP (Goals and Development, Logistics and Operations, Team and Culture, Coaching and Selection) as dependent variables was used to analyze the data.

## Results

### Organizational stressors prevalent among football players and coaches in the Ghana premier league

The results in Table 1 show that generally, stressors were common among premier league coaches (*M* = 3.36, *SD* = 2.77) and players (*M* = 2.77, *SD* = 2.77). The intensity level of stressors experienced was moderate and coaches significantly experienced it more than the players. Focusing on specific areas of stressors, both players and coaches experienced them in very similar areas of their work. For example, the coaches experienced high-stressor demands for selection (*M* = 3.55, *SD* = 1.15), team and culture (*M* = 3.47, *SD* = 0.85) and goals and development (*M* = 3.45, *SD* = 0.78). There were moderate demands for two stressor indicators for coaches, namely, logistics and operations (*M* = 3.23, *SD* = 0.99), and coaching (*M* = 3.10, *SD* = 1.41).

**Table 1:** Descriptive statistics (means and standard deviation) on the prevalence of organizational stressors among football coaches and players.

**Indicators**	**Coaches**		**Players**		***t*** **-value**	***p*** **-value**
	**Mean**	**SD**	**Mean**	**SD**		
Goals and development	3.45	0.78	2.81	0.99	4.99	0.000
Logistics and operations	3.23	0.99	2.72	1.03	3.27	0.002
Team and culture	3.47	0.85	2.73	1.14	5.28	0.000
Coaching	3.10	1.41	2.64	1.48	1.99	0.047
Selection	3.55	1.15	2.96	1.48	3.11	0.012
Mean of mean	3.36	0.81	2.77	1.02	4.46	0.000

A similar trend of results was found for the players. Although the football players reported moderate intensity levels on all the stressor indicators, team and culture (*M* = 3.47, *SD* = 1.14), selection (*M* = 2.96, *SD* = 1.48), as well as goals and development (*M* = 2.81, *SD* = 0.99) were the most dominant stressor demands for the players. This trend was followed by logistics and operations (*M* = 2.72, *SD* = 1.03) and coaching (*M* = 2.64, *SD* = 1.48).

### Contribution of age and years of experience on organizational stressors among premier league players and coaches

This research question sought to examine the contribution of age and years of experience to organizational stressors among football coaches and players in the premier league in Ghana. In order to meet the minimum requirement on the number of respondents in each cell, the following categorisations were done: (1) ages of coaches were categorized into <35 and 35 years and above, (2) ages of players were transformed into <25 and 25 years and above, (3) years of experience for both coaches and players were classified into 5 years and less, and more than 5 years. These categorisations were done to ensure adequate cell sizes and to get useful sample split for analyzing the data (Pallant, 2020). Prior to the analysis, the key assumptions: univariate and multivariate normality, outlier detection, multicollinearity, and homogeneity of variance-covariance matrices were tested and found satisfactory. There was no violation of the homogeneity of variance-covariance assumption for both players and coaches. Therefore, Wilk's Lamda estimates was reported.

### MANOVA results for players on the contribution of age and years of experience on organizational stressors

Table 2 presents the multivariate results of the MANOVA analysis for players. The results showed that experience [*F*(5, 414) = 0.502, *p* = 0.775], age [*F*(5, 414) = 0.522, *p* = 0.760], and experience-by-age interaction [*F*(5, 414) = 1.046, *p* = 0.390] did not significantly influence the composite organizational stressor indicators among players.

**Table 2:** Multivariate results for players on the contribution of age and years of experience on organizational stressors.

**Effect**	**Value**	***F***	**df**_1_	**df**_2_	**Sig**.
Intercept	0.306	187.461c	5	414	0.000
Experience	0.994	0.502c	5	414	0.775
Age	0.994	0.522c	5	414	0.760
Experience * Age	0.988	1.046c	5	414	0.390

Dependent Variable: Organizational Stressor Indicators.

df_1_ means Degree of freedom; df_2_ means Degree of freedom 2.

Table 3 further presents the univariate results of the MANOVA analysis for the players. For the interpretation of the univariate results, a similar stringent alpha (i.e., Bonferroni correction) was also set to control for Type 1 error. The same previous procedure was followed to generate a new alpha value of 0.01.

**Table 3:** Test of between-subject effects for players on the contribution of age and years of experience on organizational stressors.

**Source**	**Dependent**	**Type III**	**df**	**Mean**	***F***	**Sig**.
	**variable**	**sum of squares**		**square**		
Intercept	Goals	842.181	1	842.181	865.381	0.000
	Logistics and operations	796.017	1	796.017	757.879	0.000
	Team and culture	763.239	1	763.239	597.653	0.000
	Coaching	772.479	1	772.479	356.322	0.000
	Selection	895.442	1	895.442	408.665	0.000
Experience	Goals	2.300	1	2.300	2.363	0.125
	Logistics and operations	1.487	1	1.487	1.416	0.235
	Team and culture	1.946	1	1.946	1.524	0.218
	Coaching	2.373	1	2.373	1.094	0.296
	Selection	1.833	1	1.833	0.836	0.361
Age	Goals	0.427	1	0.427	0.439	0.508
	Logistics and operations	0.821	1	0.821	0.781	0.377
	Team and culture	2.747	1	2.747	2.151	0.143
	Coaching	3.365	1	3.365	1.552	0.214
	Selection	0.992	1	0.992	0.453	0.501
Experience * age	Goals	0.055	1	0.055	0.056	0.812
	Logistics and operations	0.001	1	0.001	0.001	0.982
	Team and culture	2.995	1	2.995	2.346	0.126
	Coaching	0.103	1	0.103	0.048	0.827
	Selection	1.564	1	1.564	0.714	0.399
Error	Goals	406.794	418	0.973		
	Logistics and operations	439.034	418	1.050		
	Team and culture	533.812	418	1.277		
	Coaching	906.191	418	2.168		
	Selection	915.895	418	2.191		
Total	Goals	3750.556	422			
	Logistics and operations	3575.395	422			
	Team and culture	3702.813	422			
	Coaching	3861.750	422			
	Selection	4641.000	422			

Dependent Variable: Organizational Stressor Indicators sub-dimensions.

The results, as shown in Table 3, revealed that experience, age and experience-by-age did not significantly influence the specific dimensions of organizational stressor indicators for players. There was no evidence that age and experience influenced the stressors of football players.

The MANOVA results for coaches are presented in Tables 4, 5.

**Table 4:** Multivariate results for coaches on the contribution of age and years of experience on organizational stressors.

**Effect**	**Value**	**F**	**df_1_**	**df_2_**	**Sig**.
Intercepts	0.066	102.256	5	36	0.000
Experience	0.825	1.530	5	36	0.205
Age	0.915	0.666	5	36	0.651
Experience*Age	0.894	0.850	5	36	0.524

Dependent Variable: Organizational Stressor Indicators.

df_1_ means Degree of freedom; df_2_ means Degree of freedom 2.

**Table 5:** Tests of between subject effects (univariate results) for coaches on the contribution of age and years of experience on organizational stressors.

**Source**	**Dependent variable**	**Type III sum of squares**	**df**	**Mean square**	***F***	**Sig**.
Intercept	Goals	279.691	1	279.691	497.945	0.000
	Logistics and operations	244.926	1	244.926	242.533	0.000
	Team and culture	276.238	1	276.238	388.989	0.000
	Coaching	197.841	1	197.841	98.584	0.000
	Selection	285.272	1	285.272	215.970	0.000
Experience	Goals	2.019	1	2.019	3.594	0.065
	Logistics and operations	0.017	1	0.017	0.017	0.898
	Team and culture	1.092	1	1.092	1.538	0.222
	Coaching	1.769	1	1.769	0.881	0.353
	Selection	0.453	1	0.453	0.343	0.562
Age	Goals	0.952	1	0.952	1.695	0.200
	Logistics and operations	0.510	1	0.510	0.505	0.481
	Team and culture	1.377	1	1.377	1.939	0.171
	Coaching	3.630	1	3.630	1.809	0.186
	Selection	2.082	1	2.082	1.576	0.217
Experience * age	Goals	0.129	1	0.129	0.230	0.634
	Logistics and operations	0.863	1	0.863	0.854	0.361
	Team and culture	0.124	1	0.124	0.174	0.679
	Coaching	3.212	1	3.212	1.601	0.213
	Selection	0.258	1	0.258	0.196	0.661
Error	Goals	22.468	40	0.562		
	Logistics and operations	40.395	40	1.010		
	Team and culture	28.406	40	0.710		
	Coaching	80.273	40	2.007		
	Selection	52.835	40	1.321		
Total	Goals	547.694	44			
	Logistics and operations	501.506	44			
	Team and culture	561.563	44			
	Coaching	509.250	44			
	Selection	609.500	44			

Dependent Variable: Organizational Stressor Indicators sub-dimensions.

Table 4 presents the multivariate results of the MANOVA analysis for coaches. The results showed that experience [*F*(5, 36) = 1.530, *p* = 0.205], age [*F*(5, 36) = 0.666^c^, *p* = 0.651], and experience-by-age interaction [*F*(5, 36) = 0.894, *p* = 0.524] did not significantly influence the stressor indicators among coaches.

Table 5 further presents the univariate results of the MANOVA analysis. For the interpretation of the univariate result, a stringent alpha (i.e., Bonferroni correction) was set to control for Type 1 error. This approach was done by dividing the alpha level by the number of dependent variables. That is, 0.05 was divided by 5 to get a corrected alpha level of 0.01. Therefore, the *p*-values are compared with 0.01 instead of 0.05 alpha value.

The results, as shown in Table 5, revealed that experience, age and experience-by-age did not significantly influence the specific dimensions of organizational stressor indicators for coaches. There was no evidence that age and experience influenced the stressors of football coaches.

## Discussion

The first objective of this study assessed the organizational stressors prevalent among premier league players and coaches in Ghana, including the relative contribution of age and years of experience. Results of this study revealed that players and coaches in the Ghanaian premier league generally experienced many stressors. The intensity of these stressors was moderate and coaches significantly experienced them more than the players. Specifically, both players and coaches experienced high to moderate stressor demands on selection, team and culture, and goals and development, respectively. Stressors regarding logistics and operations, and coaching were the least experienced by both players and coaches in the Ghana premier league. The current findings on the prevalence of organizational stressors among football players and coaches in this study corroborates existing literature (e.g., Fletcher and Scott, 2010; Arnold and Fletcher, 2012a,b; Didymus and Fletcher, 2014; Arnold et al., 2016) on the stressful nature of the sporting environment within which athletes and coaches operate.

The finding that the Ghana premier league players and coaches experienced stressors in their work is similar to the findings of other studies conducted in the United States of America (USA), United Kingdom (UK), Australia and Europe (Hanton et al., 2005; Thelwell et al., 2008, 2016a; Mellalieu et al., 2009; Fletcher and Scott, 2010; Kristiansen et al., 2012; Thelwell, 2012; Didymus, 2017), and South Africa (Kubayi et al., 2018). For example, Didymus (2017) found that coaches experienced many stressors in relation to selection, athlete concerns, coaching responsibilities, expectations, interference, preparation, organizational management, and performance among others. Further, Kristiansen et al. (2012) examined U.S. professional soccer players' organizational stressors and related coping and found that players experienced numerous stressors relating to league and team structure, coach-athlete interaction, travel demands among others. The authors indicated that football coaches are the ones who usually set standards and control all activities in the team. Additionally, Fletcher and Scott (2010) and Kubayi et al. (2018) suggested that there is a high demand on coaches concerning what they do on key matters such as squad and team selection, athletes' wellbeing and performance, organization and administrative tasks while they try to improve their own performances (Gould et al., 2002). Similar evidence was revealed in Thelwell et al.'s (2016a) study on the perceptions of athletes on stress experienced by their coaches in elite sports environments. The authors indicated that coaches who operate at the highest competitive level perform their duties in a pressurized, complex and dynamic environment, placing a heavy demand on them to perform even in the midst of all challenges. The similarities in the findings could be as a result of standardization of the competing environment with increasing demands across professional leagues eliciting enormous burden on players and coaches. The practices, philosophies, principles, and rules governing football globally are consistent and as such similar soccer environments exist irrespective of the country. Thus, the demands exerted by supporters and management on their teams to perform well in the league, coaching practices, and coaching systems among others increase the stressor demands of both players and coaches (Kroshus et al., 2015).

Taken together, the overall supervisory role often given to the coaches to ensure that the team performs to the best of its ability at all competitions or matches has varied elements, including selection (e.g., who gets selected), team and culture (e.g., work climate) as well as goals and developmental (e.g., design of appropriate training schedule) related issues. Players, however, are expected to implement or execute the plans outlined by their coaches in pursuit of successes in matches or competitions. Hence, coaches are more likely to be burdened more compared to their players. Alternatively, whereas players are not sacked for poor performance, coaches are usually relieved of their official duties or sacked for the overall performance of their teams even though there could be multifaceted factors (e.g., caliber of players, logistics) that may not have directly been caused by coaches. Within football administration, club owners or Board of Directors (BODs) usually set performance standards that have to be met by coaches on their assumption to post and such standards put enormous demand on the coaches. Fletcher and Scott (2010) argued that a coaches' continuous employment was highly dependent on their immediate willingness to perform successfully. Therefore, performance expectations often heighten the burden on the coaches and put enormous demand on them to deliver or achieve targets set by the clubs (Didymus, 2017). There have been several instances where highly respected football coaches with rich coaching experiences have lost their jobs because of failure to meet specific performance targets set by club owners (Gould et al., 2002; Didymus, 2017; Akenteng, 2019).

Taking the selection indicator, for instance, coaches reported being burdened on how selection should be done for matches whereas players reported being pressured on how to get selected into the team for matches. For coaches, lack of clear selection criteria and procedures, selecting players for their respective positions and rotating them for matches to meet the demands of the league competition to attain favorable outcomes throughout the season could be stressful or burdensome (Didymus, 2017). Similarly, for players, the demands of playing and meeting technical and/or tactical demands, coupled with occasional squad rotation and positions are characterized with intense pressure culminating in high experiences of stressors (Fletcher et al., 2006; Didymus, 2017). Additionally, it is possible that having equally competent teammates playing similar positions and having a poor interpersonal relationship with the coaching staff could equally place high demands on players regarding selection. This argument is consistent with that of Mellalieu et al. (2009) who in their study found position security and rivalry among elite sport performers who were preparing for competition. For the athletes, although selection was a grave concern and caused moderate stressor intensity, these demands were dependent on the need to excel to attain or maintain position and/or selection (Mellalieu et al., 2009; Didymus, 2017). For teams to successfully win their matches, there is the need for coaches to make appropriate team selection that can equally match up to the standards of their opponents at any particular point in time. Further, due to performance related expectations, coaches and analogous staff are under continuous scrutiny by club owners and other stakeholders (e.g., team supporters, media) on technical, tactical and management related decisions. Hence, the fear of losing the coaching job or having contract terminated triggers and/or increases coaches' experiences of stressors (Noblet and Gifford, 2002; Mellalieu et al., 2009; Kristiansen et al., 2012; Didymus and Fletcher, 2014).

There appears to be a competing interest for both players and coaches about the selection dimension. Whereas, players would want coaches to select them for matches, coaches would also want players they have selected to put up excellent performances in order to justify or guarantee their continuous selection in the team. This situation places greater demand on both coaches (in terms of making a good selection) and players (in terms of being selected). This selection dilemma further creates a scenario where coaches see squad selection and rotation as potential stressors to them (Gould et al., 1993; Noblet and Gifford, 2002; Giacobbi et al., 2004; Hanton et al., 2005; Mellalieu et al., 2009) and players also seeing their coaches as potential stressors (Olusoga et al., 2009; Fletcher and Scott, 2010; Kristiansen et al., 2012; Thelwell, 2012; Thelwell et al., 2016a). This trend brings to the understanding of how coaches feel when players they have selected do not consistently perform well, and likewise, how players feel when they are not usually selected for matches. Although team selection causes stress for both players and coaches, coaches experienced more demands than players on the issue of team selection perhaps due to the fact that coaches immediately after selection do not have control over the player's performance as asserted by Thelwell et al. (2008). Other studies (e.g., Alsentali and Anshel, 2015) agree that a myriad of stressors that athletes experience have a linkage with their coaches. Thelwell et al. (2016a)'s investigation on the perceptions of athletes regarding their coaches' stress encounters, revealed that the coaching environment as well as the athletes themselves were adversely affected when their coaches experienced stress. Olusoga et al. (2010) also asserted that coaches believe that their athletes could be the victims of their negative stressful experiences.

Further, the study also found that both players and coaches experienced stressors in the pursuit of their goals in their career and also to develop from one level to another. This finding supports previous studies suggesting that society positively acknowledges individuals who work hard or put in maximum effort to achieve their goals whereas giving up on a set goal is viewed as a sign of weakness (Ntoumanis et al., 2014). Similarly, Heckhausen et al. (2010) suggested that goals structure people's lives and promote positive behaviors that eventually improves their wellbeing. Staufenbiel et al. (2015) reiterated that setting goals is an effective tool for performance enhancement. There is also a documentation that athletes may set goals, such as achieving personal best in teams, winning a particular event, beating a particular opponent whereas coaches' goals may be continuously winning matches to receive accolades. However, these goals may at some point not be achievable nor attainable, and can generate sustained pressure leading to stress (Lazarus, 1999; Ntoumanis et al., 2014). Most often, players and coaches set goals or targets that they need to achieve at every stage of their career (Senécal et al., 2008). Thus, they face several degrees of stressors in their quest to achieve such tasks and process goals as well as short-term and long-term goals. Similarly, players and coaches are expected to make progress or achieve their process goals from time to time as they train or learn the craft of coaching. Extant literature (e.g., Weinberg, 2010; Smith et al., 2014) has confirmed the positive outcomes of setting goals against task in the sport context for both players and coaches. For instance, short-term and long-term goals are usually set by coaches with the aim of achieving success for their teams (Burgess and Naughton, 2010; Paradis and Martin, 2012). However, coaches' inability to explicitly set specific, measurable, attainable, realistic and time-bound goals may put a significant burden on them (Nicholls et al., 2016; Weintraub et al., 2021). Additionally, players who seem not to have developed after several training sessions or achieved their personal goals for their upcoming competitions due to time limitations, injury or biological capabilities may be dropped in matches or competitions until they prove otherwise. This situation places a huge demand on them to keep working hard (Ntoumanis et al., 2014). Indeed, realizing one's inability to achieve her or his goals can be a potential stressor because stress occurs when a person's goals become unattainable (Lazarus, 1999; Ntoumanis et al., 2014).

Similarly, the team atmosphere and culture presented some level of stressor intensity for the coaches and players in the current study. For example, it is possible that the players and coaches are burdened with certain undesirable attitudes (such as rivalry among players who play the same position, exhibition of lackadaisical attitude toward attendance to training sessions and lateness) that are displayed by some teammates and other officials (Mellalieu et al., 2009). For example, players who played the same position in a game saw each other as rivals when they were preparing for a competition (Mellalieu et al., 2009). Stressing further on position security and rivalry among US professional soccer players, Kristiansen and Roberts (2010) in their study, found a participant indicating that “if two guys are competing for the same spot, they are not going to be best friends.” This situation could lead to teammates frustrating, intimidating or making their perceived rival counterparts unpopular and look incompetent in order to win the coaches' heart. Didymus (2017) further found in her study involving international and Olympic level coaches' stressors, appraisals and coping attitudes such as disrespectful behaviors, attending training with hangovers, abusing drugs, and denying mistakes as well as reporting to training grounds late or not reporting at all for training led to tensions and lack of cohesion within the team. Possibly, such attitudes can trigger unpleasant reactions from coaches and among players if care is not taken. Differences in the beliefs of players and coaches demand extra efforts by both coaches and players to reach a consensus regarding some practices or routines that they need to perform, which may also create a hostile team atmosphere. Scholars (e.g., Hanton et al., 2005; Mellalieu et al., 2009; Olusoga et al., 2009; Kristiansen et al., 2012) reiterate that team culture and atmosphere potentially affect the way players and coaches feel, think and behave. Hence, negative team atmosphere will negatively affect the feelings and thought patterns of players and coaches as well as the way they behave toward one another. This tendency is likely to cause stress for both players and coaches if not managed appropriately (Fletcher and Scott, 2010).

Results regarding the contribution of age and years of experience on organizational stressors of participants revealed that there was no interaction between age and years of experience on the organizational stressors of coaches and players. This finding is quite surprising and defies already established knowledge that age and years of experience significantly influence affect experiences of sport performers (Hagan et al., 2018). Differences in cultures may have potentially affected the current findings in this study. Besides, the sample size and within cell samples for both coaches and players appear insufficient and thus could affect the power of the statistical procedure used (Pallant, 2020). Taking the coaches, for example, only 44 of them participated in the study. This sample resulted in some of the categories having a small sample cell size. Second, the “football age” phenomenon in football can account for such a result. The “football age” phenomenon happens when football players reduce their original age to appear young. Tosam (2015), for example, argues that players usually cheat about their actual age by providing a reduced age, a typical behavior of many African athletes. Although efforts were made by the researcher to obtain the real ages of the players, it was unclear whether the players provided their real ages or “football ages.” A closer look at the demographic data, for instance, show that the majority of the players were 20 years and below. Some of these players indicated that they have 5 years of experience in the premier league. Such inconsistencies can explain why the results show a non-significant difference in stressors with regards to age and years of experience. Moreover, most players and coaches are employed on contract basis for 1 year or at most, 2 years. These contracts are renewed only based on performance. Therefore, it is unlikely that especially coaches would coach one team for many years except in few instances (Akenteng, 2019).

### Limitations

This research has some limitations. For instance, only males playing only one sport (football) in the 2020/2021 premier league season participated in the study and so findings can only be generalizable to male footballers and coaches in the 2020/2021 Ghana premier league. Further, compared to players, the number of coaches were very few and this may have affected the cell sizes used in analyzing the data. This can pose a potential threat to the results of the present study. In addition, since the prevalence and intensity of stressors may fluctuate due to the competitive nature of the league at various stages of the season, it is possible that the retrospective (e.g., “demand experienced one month ago”) and cross-sectional nature of the research may not accurately reflect the temporal variation of stressors experienced by players and coaches. Only the intensity dimension of the OSI-SP instrument was used, hence findings should be interpreted with caution. Some of the data could not be collected face to face hence, it is possible some may have just ticked without paying attention to specific details. Using the reductionist perspective and studying stressors as a unitary construct did not account for the entire stress process (e.g., stressors, appraisals utilized, responses, coping mechanism adopted and their effects on the individual; Baldock et al., 2021).

### Practical implications

Football players and coaches experience a variety of stressors within their working environments. The study revealed that interventions aimed at helping football players and coaches in stressful situations should be geared toward managing issues related to selection, team and culture and goals and development as these were found to be the most reported organizational stressors, they encountered during the 2020/2021 premier league season. This finding has implications for applied practice and research. For applied practitioners, interventions involving the use of a standard guidelines or performance-related indicators that are clear for selection for both players and coaches in the discharge of their duties might help to address or minimize their stressor demands. Further, a selection procedure that is based on objective with minimal subjective assessments on performance-related indicators should be developed by the clubs in the Ghana premier league to provide some autonomy to these coaches to control certain pertinent decisions regarding selection. Considering team and culture as a stressor, club owners should adopt mechanisms, such as good channels and skills of communication, conflict resolution benchmarks and ensuring fair and transparent procedures within the team would promote group and team dynamics. Club owners should also listen to plights and concerns of their employees to develop consensus; provide players and coaches with a strong support base and build a strong team cohesion. Regarding goals and development as a stressor, club owners, and sport psychologists could help develop a goal-setting framework that promotes respect for training and development for the players and coaches by using Specific, Measurable, Achievable, Realistic and Time bound (SMART) goals. Additionally, as a matter of preventive intervention, it will be prudent for owners of clubs to assist players and coaches by providing adequate and standard facilities and resources (both human and material) that can help them to train adequately to achieve their aims. Such interventions can be done with the help of sport psychologists through educational and career development workshops, skills development and assessment programs. Assertive training, time management training, informal and formal group conversations can all be used to facilitate wellbeing and reduce the intensity of stressors experienced by players and coaches (Frey, 2007; Cassidy et al., 2009). Future studies could target players and coaches in the professional women's league and assess what coping strategies are utilized by these professionals.

## Conclusion and recommendations

Football players and coaches who participated in the 2020/2021 GPL season experienced moderate intensity of stressors on selection, team and culture, and goals and development. Besides, coaches experienced these stressful situations higher than the players. Therefore, the Ghana premier league supervising body (i.e., GFA), in conjunction with club owners, should organize regular educational workshops and seminars, develop a framework to guide selection, incorporating specific criteria and procedures that are objective, clear and standard for both players and coaches. Creating awareness of these procedures would promote transparency, trust and confidence between players and coaches that will boost team climate, culture and thus, minimize uncertainty and insecurity. Again, owners of premier league clubs should avoid direct and indirect interferences on performance-related decisions, especially on selection issues. Such decisions should be the preserve of coaches and the technical team, whose job security may be at stake because of performance related outcomes. Opportunities should also be created for players and coaches to professionally develop themselves through relevant courses by providing scholarships and sponsorships for self-development through local and international collaborations. Moreover, due to the cross-sectional nature of this research and fewer coaches used, it is possible that the true experiences of stressors experienced by players and coaches could not be fully ascertained. Therefore, future studies should consider conducting more longitudinal research with larger sample sizes to determine how stressors among players and coaches prevail over time. Future studies should also consider using theoretical frameworks that account for the entirety of the stress experiences of participants used in stress-related research for more extensive understanding of the stress process.

## Data availability statement

The raw data supporting the conclusions of this article will be made available by the authors, without undue reservation.

## Ethics statement

The studies involving human participants were reviewed and approved by Institutional Review Board of the University of Cape Coast (IRB-UCC) approved the conduct of the study with reference number UCC/IRB/A/2016/794. The patients/participants provided their written informed consent to participate in this study.

## Author contributions

MSS and JH conceived the idea. MSS performed the analysis. MSS, JH, PO, and TS prepared the initial draft of the manuscript. All authors thoroughly read and approved the final version of the manuscript.

## Funding

The authors sincerely thank Bielefeld University, Germany for providing financial support through the Open Access Publication Fund for the article processing charge.

## Conflict of interest

The authors declare that the research was conducted in the absence of any commercial or financial relationships that could be construed as a potential conflict of interest.

## Publisher's note

All claims expressed in this article are solely those of the authors and do not necessarily represent those of their affiliated organizations, or those of the publisher, the editors and the reviewers. Any product that may be evaluated in this article, or claim that may be made by its manufacturer, is not guaranteed or endorsed by the publisher.
